# Understanding the Usage of Content in a Mental Health Intervention for Depression: An Analysis of Log Data

**DOI:** 10.2196/jmir.2991

**Published:** 2014-01-31

**Authors:** Julia EWC Van Gemert-Pijnen, Saskia M Kelders, Ernst T Bohlmeijer

**Affiliations:** ^1^University of TwenteDepartment of Psychology, Health and TechnologyEnschedeNetherlands

**Keywords:** mental health, depression, Web-based intervention, acceptance commitment therapy

## Abstract

**Background:**

Web-based interventions for the early treatment of depressive symptoms can be considered effective in reducing mental complaints. However, there is a limited understanding of which elements in an intervention contribute to effectiveness. For efficiency and effectiveness of interventions, insight is needed into the use of content and persuasive features.

**Objective:**

The aims of this study were (1) to illustrate how log data can be used to understand the uptake of the content of a Web-based intervention that is based on the acceptance and commitment therapy (ACT) and (2) to discover how log data can be of value for improving the incorporation of content in Web-based interventions.

**Methods:**

Data from 206 participants (out of the 239) who started the first nine lessons of the Web-based intervention, Living to the Full, were used for a secondary analysis of a subset of the log data of the parent study about adherence to the intervention. The log files used in this study were per lesson: login, start mindfulness, download mindfulness, view success story, view feedback message, start multimedia, turn on text-message coach, turn off text-message coach, and view text message. Differences in usage between lessons were explored with repeated measures ANOVAs (analysis of variance). Differences between groups were explored with one-way ANOVAs. To explore the possible predictive value of the login per lesson quartiles on the outcome measures, four linear regressions were used with login quartiles as predictor and with the outcome measures (Center for Epidemiologic Studies—Depression [CES-D] and the Hospital Anxiety and Depression Scale—Anxiety [HADS-A] on post-intervention and follow-up) as dependent variables.

**Results:**

A significant decrease in logins and in the use of content and persuasive features over time was observed. The usage of features varied significantly during the treatment process. The usage of persuasive features increased during the third part of the ACT (commitment to value-based living), which might indicate that at that stage motivational support was relevant. Higher logins over time (9 weeks) corresponded with a higher usage of features (in most cases significant); when predicting depressive symptoms at post-intervention, the linear regression yielded a significant model with login quartile as a significant predictor (explained variance is 2.7%).

**Conclusions:**

A better integration of content and persuasive features in the design of the intervention and a better intra-usability of features within the system are needed to identify which combination of features works best for whom. Pattern recognition can be used to tailor the intervention based on usage patterns from the earlier lessons and to support the uptake of content essential for therapy. An adaptable interface for a modular composition of therapy features supposes a dynamic approach for Web-based treatment; not a predefined path for all, but a flexible way to go through all features that have to be used.

## Introduction

Web-based interventions for mental health treatment are promising. Clients prefer these treatments to face-to-face treatments alone, and Web-based interventions are successful in realizing the objectives of treatment [1-4]. Such interventions can be considered effective in access to and reduction of mental complaints comparable to traditional face-to-face therapy [2], although the reach of interventions is quite biased; most users are female and highly educated. To understand the effects of Web-based interventions, it is important to know which elements of an intervention contribute to effectiveness [2]. Many studies focus on whether and which user characteristics explain or predict the effects of an intervention, thereby overlooking the characteristics of the Web-based intervention that can influence the effects. From prior studies we know that interactivity (eg, support from a counselor [5]), dose-response, frequent updates of content, and persuasive technology (eg, reminders) increase adherence to a Web-based intervention [6].

Adherence, the usage of a system as intended, appears to be a mediator for realizing treatment objectives. In this connection, log data are important to get objective and real-time information about the usage of the intervention, how users “walk through the intervention” when they log in, and what actions they carry out during the login period. Log data as such can provide a broad and in-depth insight into adherence or (non)adherence during a treatment process. The number of logins, the number of actions per participant, and the proportion of completed modules (lessons, actions) and time spent in the treatment course have been used as metrics to identify the attrition curve and the differences between user groups, for example adherers, nonadherers, or lower active users [7]. A higher number of completions of activities or modules per login led to a greater benefit of the treatment program than little login activity or medium activity per login [7]. This can imply that support for login can improve outcomes.

Recent studies have shown that adherence to a Web-based intervention can be predicted on the usage patterns of an intervention [8,9]. For example, the number of logins in the first week of a treatment process or the number of active days in the first week can be used as predictors for adherence to the interventions. Understanding how users interact with technology is important to increase the ease of using a Web-based intervention and to avoid dropouts. In particular, log data from the early usage of the intervention can provide valuable prompts for employment of persuasive triggers to remind low-active users to login. For example, log data from a lifestyle (weight loss) Web-based intervention that combines social networking tools (blogs, discussion forum) with an online dietary intervention program [8] showed that high usage of the intervention during the first weeks (early interaction) was highly correlated with retention of the user to the intervention. Usage was a stronger predictor for a return than behavior intentions or demographics. The usage of persuasive features, such as social support, influenced the return and adherence to the intervention.

To improve the ease of use and persuasiveness of the Web-based interventions, insight is needed into not only the number of logins or proportion of completed modules but also the usage of the features of the intervention over time. The study [9] used log data to understand whether high and low adherers differ in the usage of the features of the intervention. The log files contained data about actions taken by each participant: user ID, action type (for example “logged in”), action specification (to know how they “walk” through the intervention, eg, lesson 1, screen 11), and which elements were “visited” (clicks on, eg, feedback), the time, and day. Based on that data, the number of times each participant performed an action in the Web-based intervention was extracted. For logins, this meant that not only the total number of logins per participant during the intervention period was extracted, but furthermore, the number of logins per participant per lesson of the intervention. From this data it was possible to observe, for each participant, which elements in the intervention (lessons) had been reached and which specific actions were undertaken (viewing feedback for example) at what moment. The emergent patterns in usage (low/high visited elements) and differences between users (low/high adherence) can be of value to know the critical moments for employment of persuasive features to support motivation, to explain the risks of dropout, and to stimulate users in a positive way to achieve their goals. The differences in user patterns can be used to motivate low or medium adherers to log in and to remind them to use the content that is crucial for progress in treatment.

To optimize a Web-based intervention, log data can be used to predict what features and what combination of features correlate with adherence and effects. From a previous study [6], we know that Web-based interventions consist of different content features such as education, information, exercises, monitoring, in combination with a variety of persuasive features, such as SMS text messaging (short message service, SMS) and reminders, to support users during the treatment process. In most cases, the content in Web-based interventions is based on therapies to change behaviors or lifestyles, similar to the traditional face-to-face behavior change interventions [2]. Research on the format or design of the content of Web-based interventions is scarce; most research is done to compare Web-based interventions with traditional interventions in terms of perceived helpfulness, satisfaction or reached goals, and effect sizes (meta-analysis) [2]. The content element for a Web-based intervention is often applied straight to a Web-based environment, usually organized in accordance with the traditional face-to-face approach, providing a scheme and schedule for doing homework, exercises, and for providing feedback. The employment of persuasive features (reminders, etc) seems rather ad hoc and intuitive [6].

Log data analysis can provide rich and worthwhile knowledge about how an intervention works in practice and which elements in an intervention (content and system) should be improved in such a way that participants can benefit more from the intervention. In particular, it is important to know how the content of a Web-based therapy should be presented to encourage participants to follow the treatment path. In particular, for content-driven interventions delivered via the Internet, the use of persuasive features, such as reminders, can be important to motivate participants to take up the education, information, and exercise modules from the therapy. This requires that log data are incorporated appropriately in the intervention in order to measure the performance in practice. To do so, a log protocol can be developed to gather information about users (login ID, informed consent), action types (login/logout, start lesson, start exercise, download exercise, view feedback message, etc), action specification (eg, name of text message that was viewed, visited page of system), time, and day [9-11].

The results of log data can be used to optimize the usability of the system and the persuasiveness of the content of the intervention. For example, when certain features are not used as expected, the log files and usage patterns can be considered for investigating problems related to ease of use, for example, features that are not well incorporated in the system and as such could be overlooked. Reminders, for example, might not be well connected to the content, which could result in no actions. As such, log data can provide the prompts for a more dynamic and flexible presentation of content for a Web-based therapy than a “self-help-book on the Internet”. Therefore, the aims of this study were (1) to illustrate how log data can be used to understand a Web-based intervention that is based on acceptance and commitment therapy (ACT) and (2) how log data can be of value for improving the incorporation of content in Web-based interventions.

This study was built on the findings of a prior study [9] using log data to identify differences in usage activities of adherers and nonadherers. The study found that the predictive value of characteristics of participants on adherence is very small and that for realizing effects the uptake of all features of the intervention is important. The aforementioned studies [7,9] found that high active users can benefit more from the intervention, but that more insight is needed in how use of a program of therapy impacts outcomes. Therefore, the focus of this study was on the usage of elements or features of a therapy-based program, that is, ACT, which is a form of cognitive behavioral therapy (CBT) rapidly being implemented in mental health care. Although the content of some exercises and education in ACT may be slightly different from CBT, the generic approach of the intervention is similar. As in CBT in general, we expect that many new Web-based interventions based on ACT will be built.

## Methods

### Parent Study and Participants

In this study, we carried out a secondary analysis of a subset of the log data of a Web-based treatment aimed at reducing depression from the Web-based “Living to the Full” intervention.

The analyses described in this paper have been performed on a subset of data collected in the parent study on the adherence of the Web-based intervention for the prevention of depression. Participants were adults with mild to moderate depressive symptoms, that is, >9 and <39 on the Center of Epidemiologic Studies—depression scale (CES-D) [12], who completed our online screening procedure. Participants were recruited from the general public (advertisement) and were selected to avoid users who are not a target of therapy. For the current study, data from participants who started the first of nine lessons were used. Therefore, we used the data of 206 out of the 239 participants of the parent study [9].

### The Web-Based “Living to the Full” Intervention

#### Context

The Web-based “Living to the Full” intervention is based on ACT [13] and mindfulness [14,15] and has been published as a self-help book [16]. The intervention has been shown to be effective in reducing depressive and anxiety symptoms as a group and self-help course with email support [17-19]. The Web-based intervention is an individually based self-help program. Participants can access the Web-based intervention at any time, from any place, free of charge. The intended usage is 3 hours per week.

ACT consists of three main processes: open, centered, and engaged responding [20]. The first process, open responding, consists of acceptance of negative emotions and feelings and cognitive defusion. The opposite of acceptance, experiential avoidance (EA), has been defined as the attempt to escape or avoid personal events (emotions, memories, thoughts), even when the attempt to do so causes psychological harm [21]. Avoidance strategies, such as the excessive use of medication, food, or alcohol, or refraining from work or social activities, are common reactions to distress and are often effective in the short term. However, in the long term, EA tends to foster feelings of frustration and escalating psychological distress. Cognitive defusion is the ability to let go of entanglement with negative thoughts by viewing them from a distance in a non-judgmental way. The second process, centered responding, consists of mindfulness. Mindfulness refers to a state of being attentive to and aware of experiences (including physical sensations, emotions, thoughts, imagery) occurring in the present moment in a non-judgmental or accepting way [14,22]. The third process, engaged responding, refers to knowing one’s personal values and to committing one’s self to actions based on these values, even in the presence of undesired feelings and thoughts [23]. From the perspective of ACT, values can be seen as an intrinsic motivating framework for leading a meaningful life. When a person is open, centered, and engaged, they are considered to be psychologically flexible, that is, able to act effectively in accordance with personal values in the presence of negative private experiences [24]. The three main processes of ACT are highly interrelated, and one cannot be psychologically flexible without responding in all three ways [20].

#### Content

**Figure 1:**
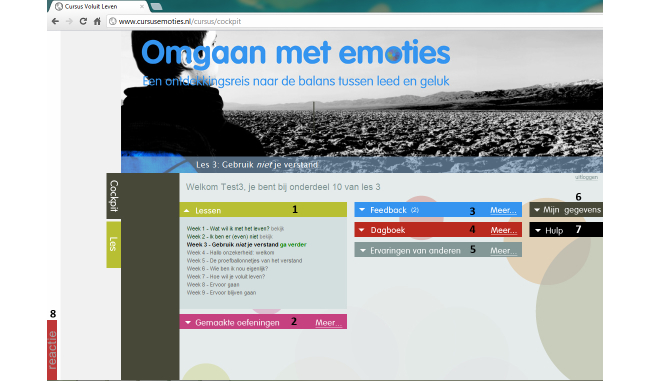
The home screen of the Web-based intervention "Living to the Full".

When participants log in to the Web-based intervention, they start in the “cockpit”. This home page of the Web-based intervention is presented in Figure 1. The Web-based intervention included a number of features, which can be grouped as general features, content features, and supportive or persuasive features. General features within the intervention are My Account (Figure 1, number 6), where participants can edit personal information as their username and password; Help (Figure 1, number 7), where participants can find basic information about the use of the intervention itself; and a React button (Figure 1, number 8), where participants can comment on the intervention. Content features are lessons (Figure 1, number 1) and exercises (Figure 1, number 2) and will be elaborated on in the next section. Persuasive features have been designed to support the uptake of content of the nine lessons and will be discussed in a following section.

#### Content Features

The ACT-based intervention consisted of nine lessons (see Figure 1, number 1) that had to be completed in a chronological order in a 12-week period. Participants were free to choose whether they worked through a lesson in one session or in multiple sessions. Participants were expected to follow all nine lessons to receive the full extent of the ACT processes.

Lessons 1, 2, and 3 focused on becoming aware of the negative consequences of EA among participants (so they became aware of the consequences of not responding openly, the first process of ACT). For example, in Lesson 2, participants made a list of all the stresses they were experiencing. They mapped all the coping strategies they used in the past in order to get control of their distress and the investments (eg, time, money) that were involved in applying these control strategies. The participants were then asked to consider the effectiveness of these strategies in the short term and in the long term and to consider that these strategies were not effective in reducing distress in the long term despite the large amount of time and money they invested. This gave rise to a feeling of creative hopelessness [13]. They became aware that the agenda of control has not been working for them, and this awareness created feelings of hopelessness. At the same time, these feelings opened up the possibility of an alternative coping strategy: acceptance of negative emotions, feelings, and thoughts. In Lessons 4, 5, and 6, participants practiced open responding. The participants learned to accept undesired emotions and to defuse negative thoughts and to disidentify with outer world aspects such as status or luxury goods and inner states such as feelings. In Lesson 5 for example, the participants had to write down five thoughts that bothered them a lot. As a second step, they had to write before each thought “I have the thought that…”. This exercise helped them to become aware that what they were thinking were mere thoughts and not reality. Another exercise was a mindfulness exercise in Lesson 4 in which they had to focus on a difficult situation they had experienced. The participants learned to notice and accept unpleasant feelings in their bodies and stay present with any negative emotions that might arise. In Lessons 7, 8, and 9, the central focus was on engaged living (third process of ACT). Participants identified important values in various life domains such as work or personal relationships and identified actions in compliance with these values. For example, in Lesson 7, participants had to clarify their core values in life domains such as relationships, work, and health. They then had to indicate to which extent they were currently living in accordance with these values. They also had to define actions that were congruent with their values. Exercises were done to strengthen the commitment to value-based living. Centered responding is a focus in all lessons.

Important transitions are between Lessons 3 and 4 and between Lessons 6 and 7. Between Lessons 3 and 4, participants had to let go of the agenda of control and avoidance and to adopt the agenda of acceptance of distress. An example of an exercise was the tug-of-war metaphor. Participants were invited to imagine themselves in a tug of war with their unwanted emotions and thoughts. This is an exhausting battle that cannot be won. As an alternative, they could decide to drop the rope and end the tug of war. Between Lessons 6 and 7, participants were invited to make decisions and change their behavior based upon their values even in the presence of unwanted feelings and thoughts. In the last three lessons, participants had to “walk the talk”.

The nine lessons were structured as follows. Each lesson started with an introduction of the topic of that specific lesson related to the aforementioned ACT and lessons. Every lesson incorporated exercises, homework, mindfulness exercises, metaphors (like the tug of war), and information related to that specific lesson. A diary could be used starting in Lesson 2. In each lesson, a short mindfulness meditation exercise was introduced that participants had to practice on a daily basis at home. In Lessons 1, 2, 5, 6, and 8, a new mindfulness exercise was presented in an online audio player, but participants had the opportunity to download the exercise as an MP3 file as well (see Multimedia Appendix 1). In Lesson 3, the participant was asked to repeat the exercises of Lessons 1 and 2. In Lessons 4 and 7, the mindfulness exercise was presented through text instruction. In Lesson 9, no new exercise was introduced. Rather, the participants were advised to integrate mindfulness exercises in their daily life. Users could start an exercise and download the exercise for practicing (see Multimedia Appendix 1).

#### Persuasive Features

The persuasive features that were available through the nine lessons for all participants were feedback (Figure 1, number 3), a diary (Figure 1, number 4), and success stories (Figure 1, number 5). Participants received feedback via the system after completing a lesson. They had to view the feedback by clicking on the feedback option (see Multimedia Appendix 1). The feedback was provided when a participant had viewed all psychoeducational material and had completed all the exercises. Furthermore, the feedback was sent to the Web-based system at least 5 days after the participant had started the lesson. This was done to ensure that a participant spent enough time on each lesson to be able to fully process the information*.* All feedback was designed to support the participant in the uptake of the content and was intended to strengthen the ACT principles.

Participants could use the diary feature to monitor their behavior and their learning of the ACT processes. The diary was not an obligatory feature of the intervention and was purely for the participant; the counselor had no access to the diary entries.

The intervention included nine success stories; after the completion of each lesson, a new success story became available for the participants (Multimedia Appendix 1). Each story had the same format in which a fictional earlier participant answered questions on the meaning of the course to them, positive experiences with the course, difficult moments within the course, and what advice they could give the current participant. Each story was different in that it focused on the content of the lesson in which the story was released. The goal of the success stories was to support the uptake of the content of each lesson and for the participants to be able to identify themselves with other users of the intervention.

Apart from persuasive features that were available for all participants, there were two features that were available for only a part of the participants. These features were part of the fractional factorial randomized controlled trial design of the parent study; both features were available for approximately half of the participants. Inclusion of these features was randomized, so a participant could have both features present, a single one, or neither of them. These features were the inclusion of multimedia (ie, video clips) within the lessons and the inclusion of an SMS coach (Multimedia Appendix 1).

For the participants in the scenario that included video, Lessons 1 to 8 included a clip in which an expert in ACT gave a thorough explanation of the ACT process central to that lesson. The videos were intended to support the understanding of the ACT processes, but the same content was also explained in the psychoeducational material available for all participants.

The participants in the scenario that included an SMS coach had the opportunity to turn on the SMS coach. This meant they received three SMS text messages per lesson on their mobile phone. Regardless of whether the SMS coach was turned on or off, for the participants in that scenario, all SMS text messages were presented within the Web-based intervention as well (Multimedia Appendix 1). The messages were intended to remind the participants to continue the lessons and to do the mindfulness exercises, but they were also intended to support the uptake of the ACT processes in daily life. This was done, for example, by posing ACT-related questions tailored to the lesson the participant was working on.

### Data Collection and Analysis

In this study, we focused on a subset of the data that was collected in the larger study. Our focus was on the usage of the content and persuasive features per participant per lesson. These data were collected by automatically logging the actions of each participant within the Web-based intervention. From these data, the number of specific actions per participant per lesson were extracted.

Of the content features, the number of logins within a certain lesson was logged. We did not log viewing of the different screens of psychoeducational material and exercises within each lesson because of the obligatory and chronological nature of each lesson (ie, all the screens in each lesson had to be viewed in order for the participant to be able to go to the next lesson). Of the mindfulness exercises, the number of times a participant started and/or downloaded each exercise was logged (Multimedia Appendix 1).

Of the persuasive features that were available for all participants, we logged the number of feedback messages viewed and the number of success stories viewed. Usage of the diary feature was not logged due to the optional nature of this feature and because the diary was purely for the participants themselves.

Of the persuasive features that were available to some participants, we logged the number of video clips viewed, the number of SMS text messages viewed within the Web-based intervention, and the number of times the SMS coach was turned on and off.

To summarize, the log files used in this study were per lesson: login, start mindfulness, download mindfulness, view success story, view feedback message, start video, turn on SMS coach, turn off SMS coach, and view text message. Action specifications were, for example, the name of the mindfulness exercise that was started or which text message was viewed and was used to identify the number of unique actions per lesson (eg, did the participant view the same feedback message multiple times or did the participant view different messages?).

Statistical analysis was done with SPSS 20. For all analysis an alpha of .05 was used as the level of significance. Differences between lessons were explored with repeated measures analysis of variance (ANOVA) where the factor “time” had nine levels (one for each lesson of the intervention). For the trend analyses, we were mainly interested in whether there was a linear trend (eg, whether during the 9 time points, the overall score increased or decreased). However, significant fluctuations over time may also be of interest. Therefore, we also investigated whether there was a quadratic, cubic, or order 4 until order 8 effect. For this paper, significant higher order effects were interpreted as a significant fluctuation in use over the nine lessons. The higher the order, the stronger the fluctuations in usage.

To explore differences between higher and lower active users, a quartile split was used to divide the participants into four groups based on the average number of logins per started lesson. Differences between these groups on usage of the different features were explored with one-way ANOVAs. Differences between the groups on the number of participants who turned the SMS coach on were explored with a chi-square test.

To explore the possible predictive value of the login per lesson quartiles on the outcome measures, four linear regressions were used with login quartiles as predictor and with the outcome measures (CES-D and Hospital Anxiety and Depression Scale—Anxiety [HADS-A] at post intervention and follow-up) as dependent variables. Depressive symptoms were measured with the CES-D (20 items, score 0-60; higher scores mean more depressive symptoms) [12,25] at baseline, post intervention, and follow-up. Anxiety symptoms were measured with the HADS-A (7 items, score 0-21; higher scores mean more anxiety symptoms) [26,27] at baseline, post intervention, and follow-up.

Missing data on clinical measures (CES-D and HADS-A) were imputed with the expectation-maximization method in PASW 18. This method estimates the unmeasured data based on maximum likelihood estimates using observed data in an iterative process [28]. Observed data on CES-D, HADS-A, gender, age, education, lesson reached, and support situation were used for estimation.

## Results

### Usage of Content and Persuasive Features per Lesson

Of the 238 participants that were randomized and received login information, 206 participants logged in (Lesson 1) and 118 participants completed Lesson 9 (Table 1). The use of the intervention (adherence rate) fluctuated over time; critical moments for dropout appeared in Lessons 3 and 6 [9].

Table 1 presents the mean login and the mean use of features that were available for all participants per lesson. Over time, the mean login activities for the features available for all participants decreased significantly: linear effect, *P*<.001, *F*_1_=13.656 (see Figure 1). Table 1 shows that not all features have been used in all the lessons, like mindfulness (unique messages, downloaded, success stories; mean score below 1).

**Table 1:** Mean logins and mean use of features available for all participants per lesson.

Lesson No. (n)	Login,	Feedback^a^, mean (SD)	Unique^b^feedback, mean (SD)	MF^c^started, mean (SD)	Unique MF started,	Unique MF downloaded, mean (SD)	Success stories, mean (SD)	Unique success stories, mean (SD)
1 (206)	4.40 (3.05)	2.04 (2.34)	1.36 (1.00)	1.75 (1.59)	0.88 (0.33)	0.50 (0.50)	1.67 (1.53)	1.28 (0.78)
2 (194)	4.12 (2.82)	2.10 (2.37)	1.53 (1.18)	1.28 (1.31)	0.78 (0.49)	0.51 (0.58)	1.03 (1.40)	0.87 (1.06)
3 (174)	3.92 (2.58)	2.20 (2.94)	1.53 (1.56)	0.83 (1.41)	0.57 (0.77)	0.03 (0.17)	0.84 (1.29)	0.68 (1.00)
4 (159)	4.27 (2.34)	2.44 (3.60)	1.68 (1.78)	0.11 (0.55)	0.06 (0.29)	0.03 (0.22)	0.73 (1.20)	0.63 (0.97)
5 (152)	3.70 (2.00)	1.99 (2.53)	1.50 (1.38)	1.08 (1.01)	0.73 (0.47)	0.40 (0.59)	0.51 (1.05)	0.44 (0.84)
6 (149)	4.26 (3.25)	1.87 (2.54)	1.46 (1.59)	1.03 (1.32)	0.64 (0.56)	0.40 (0.63)	0.58 (1.28)	0.52 (1.08)
7 (135)	3.96 (3.79)	2.29 (3.36)	1.73 (2.09)	0.05 (0.45)	0.02 (0.15)	0.06 (0.40)	0.76 (1.76)	0.58 (1.27)
8 (125)	3.54 (2.90)	2.99 (6.58)	1.98 (3.11)	0.82 (1.13)	0.58 (0.73)	0.50 (0.75)	0.94 (2.31)	0.76 (1.59)
9 (118)	3.95 (4.22)	2.97 (3.97)	2.19 (2.61)	0.23 (1.03)	0.15 (0.63)	0.34 (1.14)	0.95 (2.10)	0.80 (1.63)

^a^Feedback messages viewed.

^b^Unique per lesson, that is, the number of different messages or exercises that a participant used in a particular lesson.

^c^MF: mindfulness exercise.

### Feedback

**Figure 2:**
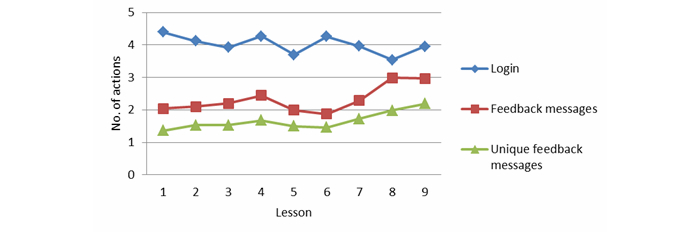
Login and feedback per lesson.

Figure 2 shows the feedback per lesson. Participants received feedback after having completed all the exercises of that lesson. The use of feedback messages did not change significantly during the treatment process. By contrast, the number of unique feedback messages viewed increased significantly during the treatment process (linear effect, *P*=.025, *F*_1_=5.176). This shows that at the beginning the participants viewed the same messages multiple times (more total messages, but not more unique or different messages), and later on, participants viewed different messages, but each of these messages fewer times (the same number of total messages, but more unique messages).

### Mindfulness

**Figure 3:**
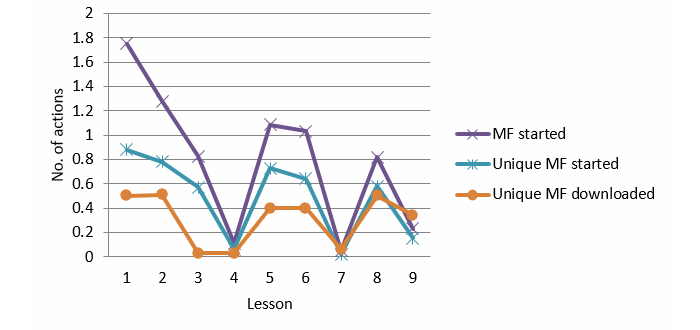
Mindfulness per lesson.

Mindfulness exercises were introduced in each lesson for daily practice. The use of mindfulness exercises decreased significantly over time: linear effect, *P*<.001, *F*_1_=99.029 (see Figure 3). Furthermore, there were differences over time, where Lessons 4 and 7 showed the least use of mindfulness exercises: quadratic (*P*<.001), cubic (*P*<.001), order 5 (*P*=.016), order 6 (*P*<.001), order 7 (*P*<.001), and order 8 (*P*=.025) effect.

There were more participants who started to use the mindfulness exercises than participants who downloaded these exercises (for offline practicing). The pattern for the usage of unique mindfulness exercises is the same. There was a significant decrease over time (linear effect, *P*<.001, *F*_1_=100.042) and a variance in usage over time: quadratic (*P*<.01), cubic (*P*<.001), order 5 (*P*=.015), order 6 (*P*<.001), order 7 (*P*<.001), and order 8 (*P*<.001) effect. Downloading of mindfulness exercises did not increase or decrease over time (linear effect, *F*_1_=0.340, *P*=.561), but there was a significant variance in usage during the treatment process: quadratic (*P*<.001), cubic (*P*<.01), order 5 (*P*<.001), order 6 (*P*<.001), and order 7 (*P*<.001) effect (see Figure 3).

Lesson 3 mindfulness exercises were repeated from Lessons 1 and 2, and Lessons 4 and 7 had only text as content for the mindfulness exercises. The use of started (linear effect, *P*<.001, *F*_1_=41.511) and unique started (*P*<.001, *F*_1_=24.975) online mindfulness exercises in Lessons 1, 2, 5, 6, and 8 decreased significantly.

A comparison of Lessons 1, 2, 3, 5, 6, and 8 showed a similar pattern for started (linear effect, *P*<.001, *F*_1_=36.787) and unique started (linear effect, *P*<.001, *F*_1_=22.485) exercises. There was a decrease of unique downloaded mindfulness exercises for Lesson 3 (order 4 and 5; *P*<.001, *F*_1_=34.152).

### Success Stories

**Figure 4:**
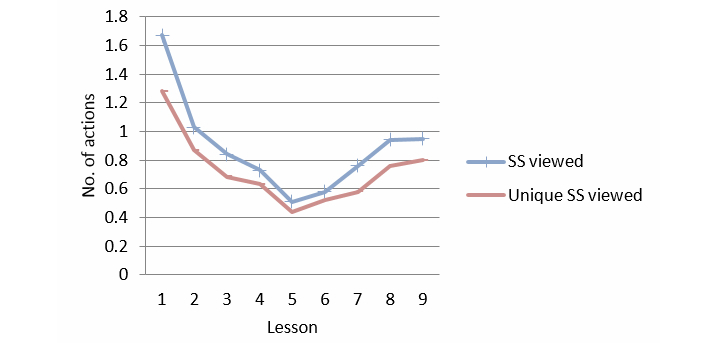
Success stories per lesson.

After completion of each lesson, a new success story was introduced. The use of success stories decreased significantly over time (viewed, linear effect, *P*<.001, *F*_1_=17.020; and unique viewed, *P*<.001, *F*_1_=13.745). A significant fluctuation over time was observed: quadratic effect, *P*<.001 for both viewed and unique viewed (see Figure 4).

### Usage of Text Messages and Multimedia per Lesson

Table 2 presents the number of participants that received the SMS and video features and the mean use of these features, per lesson. The SMS coach was available in the Web-based system, and participants were also able to turn on the SMS coach on their mobile phone. All SMS text messages were presented within the Web-based system, regardless of whether the mobile phone option was activated or not. Table 2 shows that only a few used the mobile phone option for SMS messages.

**Table 2:** Mean use of features, per lesson for the features SMS and video.

Lesson No.	SMS feature^a^, n	SMS on^b^, n	SMS off^b^, n	SMS viewed^c^, mean (SD)	Unique SMS viewed^d^, mean (SD)	Video feature^a^, n	Videos viewed, mean (SD)	Unique videos viewed, mean (SD)
1	105	13	3	1.44 (2.35)	0.95 (1.27)	116	0.59 (0.94)	0.40 (0.49)
2	98	2	0	1.32 (2.43)	0.99 (1.62)	109	0.44 (0.87)	0.28 (0.45)
3	85	0	0	1.09 (2.29)	0.86 (1.68)	96	0.61 (1.32)	0.39 (0.49)
4	79	0	0	1.54 (3.39)	1.19 (2.39)	91	0.74 (1.05)	0.45 (0.50)
5	74	0	0	0.96 (2.62)	0.84 (2.19)	90	0.66 (0.96)	0.46 (0.54)
6	73	1	0	1.21 (3.86)	0.99 (2.55)	87	0.49 (0.54)	0.40 (0.56)
7	71	1	0	0.86 (2.26)	0.68 (1.80)	79	0.66 (1.32)	0.42 (0.52)
8	66	0	0	2.44 (2.14)	2.14 (5.95)	71	0.66 (0.99)	0.48 (0.61)
9	63	0	2	1.92 (6.05)	1.48 (4.56)	64	0.05 (0.38)	0.03 (0.25)

^a^Number of participants who received the intervention with the feature included.

^b^Number of participants who turned the SMS coach on/off in a particular lesson.

^c^Number of SMS messages viewed within the Web-based intervention.

^d^Unique per lesson, that is, the number of different messages that a participant used in a particular lesson.

### SMS Coach

**Figure 5:**
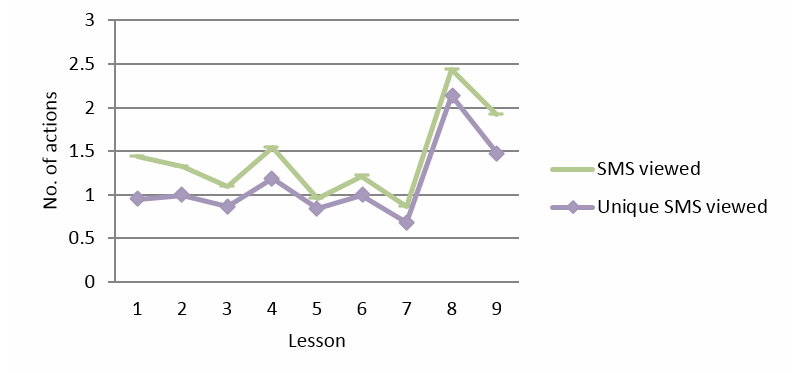
SMS per lesson.

The SMS coach provided messages per lesson to remind participants to do the exercises and to support them via tailored messages during the lessons. Figure 5 shows the viewed and unique viewed SMS messages, per lesson. The number of viewed SMS messages over nine lessons did not increase or decrease over time. However, there was a significant fluctuation in usage: SMS viewed (linear effect, *P*=.579, *F*_1_=0.311; quadratic effect, *P*=.032; and order 8 effect, *P*=.018) and unique SMS viewed (linear effect, *P*=.274, *F*_1_=1.219; and order 8 effect, *P*=.004).

### Multimedia

**Figure 6:**
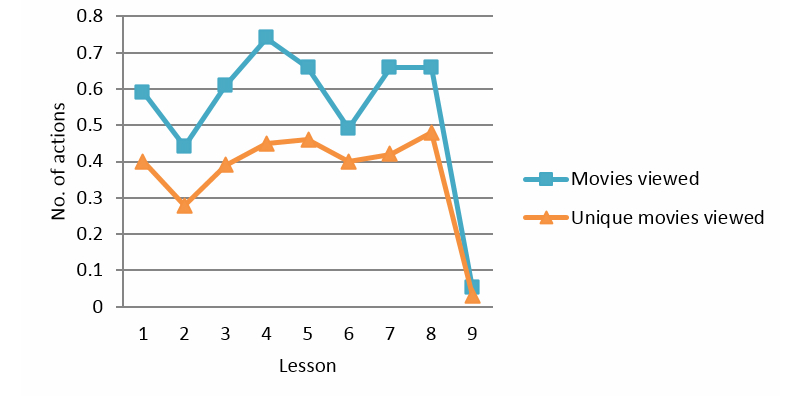
Movies per lesson.

Videos to explain the treatment and to understand the content of a particular lesson were provided from Lessons 1 to 8. The videos viewed (linear effect, *P=*.01, *F*_1_=7.133) and unique videos viewed (linear effect, *P*<.001, *F*_1_=15.935) over nine lessons showed a significant decrease and a fluctuation in usage (quadratic) over time (videos viewed: quadratic effect, *P*<.001; order 5 effect, *P*<.01, and unique videos viewed: quadratic effect, *P*<.001; cubic effect, *P*<.001; order 4 effect, *P*=.041; order 5 effect, *P*<.01) (see Figure 6). Without Lesson 9 (because there was no video for Lesson 9), there was no significant decrease in videos viewed and unique videos viewed (videos viewed: linear effect, *P*=.977, *F*_1_=0.001; and unique videos viewed: linear effect *P*=.244, *F*_1_=1.382). Only a difference in usage over time was observed for unique videos viewed (order 4 effect, *P*<.1).

### Use of Content and Persuasive Features for Different Users

Table 3 shows the average actions per started lesson (mean use of features), by level of logins per started lesson (quartile of logins). The average number of logins per started lesson was used as a measure to divide users into higher (3^rd^, 4^th^quartile) and lower active users (1^st^, 2^nd^). Higher logins over time (9 weeks) corresponded with a higher usage of features (in most cases significant; see Table 3) and vice versa.

**Table 3:** Mean logins and mean use of features, for different users (1^st^-4^th^quartile, n=206).

	1^st^quartile logins (n=51)	2^nd^quartile logins (n=57)	3^rd^quartile logins (n=49)	4^th^quartile logins (n=49)	*F* (df)	*P*
Login, mean (SD)	1.95 (0.46)	3.18 (0.31)	4.24 (0.38)	6.55 (2.30)	139.376 (3, 202)	<.001
Feedback, mean (SD)	0.97 (0.77)	1.63 (1.07)	2.19 (1.62)	3.34 (2.36)	20.867 (3, 202)	<.001
Unique feedback, mean (SD)	0.75 (0.43)	1.08 (049)	1.17 (0.56)	1.54 (0.71)	17.090 (3, 202)	<.001
MF started, mean (SD)	0.74 (0.66)	0.75 (0.53)	0.95 (0.74)	1.15 (0.71)	4.464 (3, 202)	.005
Percentage MF started, mean (SD)	70.1 (34.7)	66.6 (30.3)	76.3 (27.7)	80.2 (30.0)	2.030 (3, 202)	.111
Percentage MF downloaded, mean (SD)	24.0 (33.0)	48.9 (40.9)	47.3 (40.1)	62.8 (40.5)	8.652 (3, 202)	<.001
Success stories, mean (SD)	0.61 (0.59)	0.92 (0.68)	0.83 (0.65)	1.29 (0.96)	7.474 (3, 202)	<.001
Percentage success stories, mean (SD)	49.1 (47.3)	61.8 (34.6)	54.9 (31.9)	70.4 (28.5)	3.189 (3, 202)	.025
SMS feature, n	25	30	25	25		
SMS turned on, %	16.0	36.7	12.0	32.0	Chi-square: 6.201 (df=1)	.102
SMS viewed, mean (SD)	0.42 (0.77)	0.73 (1.04)	1.36 (1.72)	2.37 (2.86)	6.111 (3, 101)	.001
Percentage SMS viewed, mean (SD)	11.8 (23.4)	18.5 (22.9)	28.1 (30.1)	41.1 (37.0)	4.992 (3, 101)	.003
Multimedia feature, n	22	34	30	30		
Videos viewed, mean (SD)	0.34 (0.54)	0.38 (0.56)	0.45 (0.53)	0.80 (0.83)	3.236 (3, 112)	.024
Percentage videos viewed, mean (SD)	24.3 (36.0)	26.5 (35.8)	35.7 (40.1)	43.4 (41.7)	1.508 (3, 112)	.216

### Usage and Impact

To explore the possible predictive value of the login per lesson quartiles on the outcome measures, we performed exploratory linear regressions with the login quartiles as predictor and with the outcome measures (depressive and anxiety symptoms on post intervention and on follow-up) as dependent variables. Tables 4 and 5 show that only when predicting depressive symptoms at post intervention, the linear regression yielded a significant model with login quartile as a significant predictor. However, the percentage of explained variance was only 2.7%.

**Table 4:** Linear regression with login quartiles as predictor.

Model	Included	B (SE)	*P*	Odds ratio (95% CI)
**Predicting CES-D at post intervention**				
	Constant	20.8 (0.90)	<.001	19.0-22.6
	Login quartile	–1.17 (0.49)	.018	–2.14 to –0.20
**Predicting CES-D at follow-up**				
	Constant	18.1 (0.95)	<.001	16.2-20.0
	Login quartile	–0.63 (0.52)	.229	–1.65 to 0.40
**Predicting HADS-A at post intervention**				
	Constant	8.16 (0.35)	<.001	7.46-8.86
	Login quartile	–0.37 (0.19)	.057	–0.72 to 0.01
**Predicting HADS-A at follow-up**				
	Constant	7.44 (0.33)	<.001	0.78-8.10
	Login quartile	–0.23 (0.18)	.210	–0.59 to 0.13

**Table 5:** Characteristics of the tested linear regression models.

Model	*F* (df=1, 204)	*P*	*R* ^2^
Predicting CES-D at post intervention	5.697	.018	.027
Predicting CES-D at follow-up	1.457	.229	.007
Predicting HADS-A at post intervention	3.660	.057	.018
Predicting HADS-A at follow-up	1.582	.210	.008

## Discussion

### Principal Results

The aims of this study were (1) to illustrate how log data can be used to understand the uptake of the content of a Web-based intervention based on ACT and (2) to show how log data can be of value for improving the incorporation of content in Web-based interventions.

The log files used in this study were per lesson: login, and use of features by means of starting a feature (click) and/or downloading a feature (click) for practicing. We were interested in which features of the intervention were used, in what way (viewing and/or downloading for practicing), and in what intensity (did the participant view the same features multiple times or did the participant view different features?). In addition, we wanted to know the impact of logins (high/low active uses) on outcomes and the impact of the features on outcomes. The results were of relevance for tailoring the incorporation of the content of the Web-based intervention to the stages of the program of therapy to reduce depression. The log data of this study showed that the uptake of the content is not quite in accordance with the underlying principles of therapy, and as such, it provides insights for improvement to the program.

Overall, the pattern that emerged from the log data showed a decrease in logins and a decrease in the use of content and persuasive features over time (nine lessons). The decrease in logins might be a learning effect; from the parent study [9] it appeared that adherers needed fewer sessions (logins) to complete a lesson at the end of the treatment than during the first lessons of the treatment program. The pattern of the usage of content and persuasive features varied significantly (quadratic, cubic, and in high order) over time, except for the usage of Feedback messages. The use of Feedback did not change over time, in contrast with the use of unique Feedback messages. This might be related to a need for tailored feedback during the end of the treatment process, when commitment to “value-based living” is the focus of therapy (Lessons 7, 8, and 9). The use of success stories fluctuated over time and increased after Lesson 5. The content of each story was tailored to the essence of a particular lesson and stage of therapy. Lessons 6 to 8 were part of a transition process to change behaviors based upon personal values for engaged living [21,24]. This may have influenced the need for stories with experiences of others and their advice. The use of SMS text messages fluctuated over time and increased at the end of the treatment process, after Lesson 7. An explanation might be that at the end of the treatment process, more SMS messages were available than in the earlier lessons.

The quadratic, cubic, and high order effects in usage were important to understanding the connection between the uptake of features per lesson and the three main processes of ACT that guide the lessons of the intervention (content). According to Hayes [24], these main processes are highly interrelated, and one has to go through all three stages. The underlying principle of the “Living to the Full” intervention is that participants benefit from therapy when they use all features related to the three main processes of ACT. In addition, the lessons must be done in chronological order. Mindfulness is the focus of all lessons and refers in particular to the centered responding process. The decline in mindfulness in Lessons 3, 4, and 7 was related to the mindfulness content in those particular lessons (repetition of Lessons 1 and 2, and text only in Lesson 7). In addition, the suboptimal use of online and offline features (downloaded mindfulness for practicing) indicated that participants did not benefit from all the mindfulness features that were available, which is relevant given that mindfulness is an integral part of the intervention and a central feature of ACT-based interventions. At the end of the course (Lessons 8 and 9), users were asked to integrate mindfulness exercises in their daily lives. This may have resulted in a more intensive use, which aligns with the increase in unique downloaded mindfulness exercises. Moreover, at the end, users received an overview of all the exercises done. After week 9, they were not allowed to return to the intervention with all features. So it may be that users wanted to complete all possible exercises at the end of the lessons because they knew they could not return to the features after week 9. Overall, the increase of persuasive features at the end of treatment, the engaged responding stage, might indicate that participants needed motivational support to comply with the “value-based-living” stage.

The log data from this study can be useful for understanding how the incorporation of content in a Web-based therapy can be improved, in such a way that participants can benefit more from the intervention. The study revealed that higher logins over time correlate with a higher usage of most features. Higher active users consume all features more than low active users, which may indicate a need to stimulate users to log in to stick to the program of therapy. The login per lesson did not predict the outcomes; only when predicting depressive symptoms at post intervention was the login a significant predictor, although the explained variance was low. This sheds light on the dose-response relationship: a higher active usage per lesson might lead to better results in terms of outcomes. When entering the use of the content and persuasive features per lesson as predictors (a separate model per lesson), none of the models significantly predicted reduction of depression. These findings questioned the importance of the uptake of *all* features or completing *all* modules to realize the objectives of the therapy. From our prior study [9], we know that adding a single feature to the intervention, except feedback, does not impact adherence or outcomes. Also, a variance in intensity of features (more extensive use of a single feature compared to a lower extensive use of that feature) did not influence the adherence or outcomes [29]. It might be that not a single feature but a *combination* of content and action-tailored persuasive features can play a supportive role *during* the intervention period, resulting in higher involvement and more learning effects. Are there certain combinations of features that predict adherence, return rates, or outcomes? Although the ACT-based therapy requires a fixed programming of lessons, the high (quadratic, cubic, high order) fluctuation in usage of content and persuasive features requires more flexibility in treatment, if we take into account differences in learning styles (eg, fewer sessions needed to complete the lessons) and allow for differences in paths and tempo when “walking through the intervention” (shorter paths and higher activities, for example).

In addition, the use of action-related persuasive features can strengthen the effects on adherence. A different study showed that exposure to an action-related persuasive feature (social support to reduce weight) influenced the persistence with the Web-based intervention [8]; not the action per se but the repercussions of the action influenced persistence [8]. These results are preliminary, but promising for Web-based interventions to tailor content and persuasive features to therapy and involved actions, for example, using online and offline exercises like mindfulness. Via the log data, we observed that participants, for example, started with viewing an exercise but never downloaded the exercise. This is relevant information for the developers of the intervention (identification of action-related triggers for extra support to download exercises). Log data are an objective registration of online usage. In combination with surveys, self-reports, and interviews during the intervention period, it is possible to gain insight into the reasons for activities carried out or not and in the evaluation of the system and content. The react button, one of the features of the intervention of this study, shows that most of the remarks were on the quality of the system (bugs, confusion about how the system works) and service (availability of intervention after completion) [29]. For improving the incorporation of content that plays a major role in therapy, insight into the capacities of persuasive features to activate or to engage users is important.

To benefit from log data, content experts (eg, therapists) have to communicate with designers in an early stage of intervention development about which features should be incorporated into the Web-based system and in which logical order (paths, combinations). A well-thought out activity log protocol that describes which features of an intervention should be logged and how log data can be incorporated in the intervention should guide this debate, in order to capture data for the purpose of identifying which combinations and paths (routing) facilitate the uptake of content and which combination of features (content and persuasive) works best for whom. From our research [29-31], we know that such a debate is fruitful but often complicated. First, the collaboration between developers and content experts is often problematic because developers and content experts use different “mental spaces” and different “vocabularies”. Second, the incorporation of log data can be laborious. Third, advanced data analytics are needed to identify and predict usage patterns. The debate involves an inquiry on several levels. At the level of content, we need to decide which features are integral parts of the therapy and lessons, what kind of support (guided, unguided) is intended, and what kind of persuasive features can motivate the uptake of content and offline exercises. At a system level, it is a matter of considering the logical order of content and persuasive features to be available during the lessons, as well as discussing flexibility in the “walking-path-route” through the system (eg, have participants go through the content in a chronological order, through all lessons once). At the level of users, what is the supposed eHealth literacy? At the level of evidence, what data should be collected to be able to identify factors that influence adherence, outcomes, and retention? These questions are relevant before the design of the intervention system starts. To guide such an early stage development debate, we developed guidelines with an accompanying toolkit [30,31] and a protocol for incorporating log data into Web-based interventions [9,11,29].

### Limitations

There are several limitations regarding this study. First, log data studies are in their infancy, and more insight is needed into real-time usage data to learn what works in practice and for whom. Some studies are available that report on the uptake of content in percentages of modules that are used (eg, [32]), and some have preliminary results about which features support persistence (eg, [8]). Advanced analysis techniques are needed to develop interventions that are dynamic and flexible. For example, machine learning techniques are needed to search for paths for success, to tailor the routing in the intervention to learning profiles, and to know how much effort is needed for success in outcomes (eg, the minimal, maximal amount of sessions to complete the therapy). Markov models might be of value to identify successful combinations of features. Furthermore, presentation techniques are needed to visualize the data in such a way that therapists and designers can discuss it. For the development of theory-driven Web-based interventions, an advanced login system should be built in the intervention to be able to gather data that meet the requirements for advanced analytics (machine learning, Markov models).

The other limitation we have is a reduced sample of participants that used the SMS coach and multimedia, due to a fractional factorial design in the prior study [9]. In this study, we used only log data to evaluate how the content and persuasive features have been used. The opinion of participants about the added value of these features has not been reported in this study; we will report on this in another study.

### Future Research

The findings of this study can be used for the development of a log protocol to enable advanced analytics to measure the uptake of content of a Web-based intervention and for improving the incorporation of content and persuasive features.

To develop Web-based interventions, a protocol should be developed that explains what kind of log files to build into the system to get data that are manageable and that make sense for usage analysis. Such a protocol can be published as part of the description of the Web-based intervention, for reasons of transparency and replications. In current studies, the use of log data is not reported comprehensively. A log protocol, for example, can include information about login (reach of an intervention), the time, moments of usage (exposure), the activities performed (which features are used), the intensity of usage (how many activities in 1 session), the description of a session (interaction time period after login, eg, 30 minutes, to avoid calculating when participants are inactive), the reactions (eg, a reaction on feedback), and so on. An example of this can be found in [11,29].

For an in-depth analysis of how participants can benefit more from the intervention and which combinations of features matter most to whom, advanced methods are needed to perform log data analyses and to recognize and predict usage patterns automatically. Pattern recognition can be relevant to guide participants through the intervention based on their usage and return patterns from earlier lessons and to support them in the uptake of content essential for therapy, like mindfulness. In a study on Web-based interventions for depression [33], further investigation was suggested into the relationship between viewing interactive content features aimed at improving engagement and the usage of other features of the program. In our study, no simple linear relationship was found between features and the use of content. To be able to reveal other nonlinear relationships, we will use more complex models or more advanced techniques (machine learning) in future research.

By means of log data, a better incorporation of content and persuasive features in the intervention is possible and a better intra-usability of content and persuasive features within the system (home page and accompanied subpages). The findings of this study revealed that the incorporation of content features and action-related persuasive features can be improved for a better connection of the therapy program with lessons and exercises, and to foster the practicing of exercises (not just viewing, also downloading for practicing). Furthermore, based on the findings, a discussion is needed between designers and content experts about the pacing of the therapy program. For example, do the participants need to go through the content in a chronological order, through all the lessons once? How should flexibility be built into the program for coping with the new behaviors and for self-reflection? Although the therapy program is obligatory, this does not imply a one-size-fits-all approach. Participants may differ in needs for support at different moments and might have different learning and e-learning styles and needs for self-reflection during a treatment process. The fluctuation in high orders (above 4) can be used to review the principles of treatment taking into account a variable and momentary use of features instead of completing lessons and exercises equally.

An interactive and adaptable interface for a modular composition of therapy features supposes a new concept of the Web-based treatment approach: not a predefined path for all but a flexible way to go through major features that had to be used to benefit from therapy. In terms of technology, such a flexible presentation of content is not a problem, but it first requires a debate among content experts and designers to review the benefits and drawbacks of such an approach and its implications for therapy (eg, online vs blended formats). The log data revealed that the integration of online and offline activities should be considered for improvements. There is a lack of balance between the started exercises and the downloaded exercises for practice. A better integration with mobile support (eg, to download exercises) and the incorporation of action-related persuasive triggers could be an option. The integration with mobile services has been addressed in a study about a Web-based program for depression because of the underuse of SMS text messages to create updates via mobile phone [33].

To identify the potential features for supporting better interaction with the system, we think the Persuasive System Design (PSD) model [34] can be valuable. The PSD model provides a taxonomy for incorporation of persuasive features into the system, to support tasks (eg, exercises), dialogue, social support, and to support the credibility of the system. These persuasive features can be tested in an experimental design (like the Multi-Phase Optimization Strategy [MOST] [35]) in order to assess the added values for the uptake of content of the Web-based intervention. This helps avoid a more intuitively based choice of persuasive features.

In the end, technology can support therapy. But to achieve success, the development of theory-driven content should be interwoven with the development of technology. The process of development of Web-based interventions and built-in log data should be described in a way that enables replications.

### Conclusions

It can be concluded that the log data are valuable for gaining insight into the usage of content of the Web-based “Living to the Full” intervention. Based on the log data, it is possible to tailor the content and persuasive features of the intervention to the main processes of acceptance and commitment therapy. To gain more detailed insight into how participants can benefit more from the intervention, advanced analytic methods are needed to identify which navigation route facilitates the uptake of content and which combination of features (content and persuasive) works best for whom.

The pattern that emerged from the log data showed a decrease in logins and a decrease in the use of content and persuasive features over time (nine lessons). The usage of content and persuasive features varied significantly (quadratic) during the treatment process. The uptake of mindfulness, which is an integral part of ACT, varied substantially during the treatment process in contrast to feedback. The usage of persuasive features like SMS, multimedia, and success stories and feedback increased during the third part of the ACT (commitment to value-based living), which might indicate that at this stage, motivational support is relevant.

To improve the uptake of content of the intervention, a better integration of lessons and persuasive features in the intervention and a better intra-usability of features within the system are needed. It is preferable to have an overarching view of the composition of features that are an integral part of the ACT intervention and features that are supportive. A well-planned activity log protocol that describes which features of an intervention should be logged and how log data can be incorporated into the intervention should be developed in the early stage of design. Such a protocol enables us to identify which navigation route in the system facilitates the uptake of content and which combination of features (content and persuasive) works best for whom.

## Multimedia Appendix 1

Web-based Living to the Full intervention.

## Abbreviations

ACT: acceptance commitment therapy
CBT: cognitive behavioral therapy
CES-D: Center of Epidemiologic Studies—depression scale
EA: experiential avoidance
HADS-A: Hospital Anxiety and Depression Scale—Anxiety
MOST: Multi-Phase Optimization Strategy
PSD: persuasive system design
SMS: short message service
