# Supplementary material for: Understanding the Usage of Content in a Mental Health Intervention for Depression: An Analysis of Log Data
Source: J Med Internet Res. 2014 Jan 31;16(1):e27. doi: 10.2196/jmir.2991 (PMC3936274; doi:10.2196/jmir.2991)
Supplement: Supplementary file 1 [file jmir_v16i1e27_app1.pdf]

Multimedia Appendix1 – Detailed description of supportive or persuasive features Web-based “living to the full” intervention

Feedback

Participants received weekly feedback. They have to view the feedback by clicking on the feedback option. Feedback messages were presented under ‘feedback’ in the personal home screen, accompanied by a picture of the care provider (Figure MA1.1). The feedback messages contain the key learning points and goal of the completed lesson; the key exercises and feedback on at least the core exercise; feedback on the mindfulness exercise; and a preview of the following lesson. Participants can view unique feedback messages and they can view feedback messages already sent to them (multiple viewed messages).

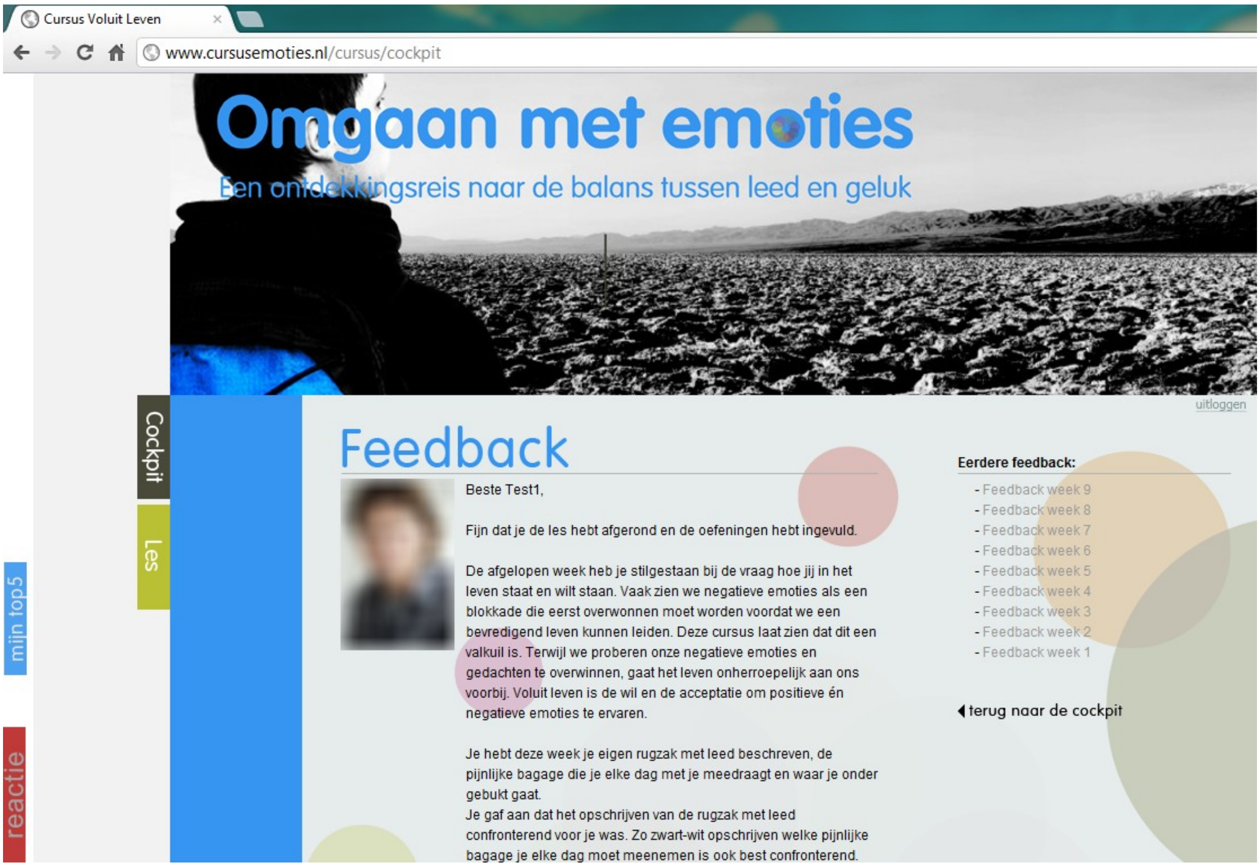

Figure MA1.1 – Feedback message Note: In this screenshot, the picture of the care-provider has been blurred for privacy.

SMS-coach(text message)

Participants had the opportunity to turn the SMS-coach on. This SMS-coach sent 3 pre-designed text messages each week to a mobile phone number provided by the participant. The timing of the text messages was different each week, but all messages were sent between

9AM and 9PM. Each week one message contained a motivational message (e.g. “Do you realize you have taken the first step to learn to ‘live to the full’? Congratulations and keep going!”), one message contained a mindfulness trigger (e.g. “How mindful are you today?”) and one message reflected on the content of that week (e.g. “Avoidance is like scratching an itch. It only works for a short time.”) All text messages were presented in the ‘text message’ tab of the application, independent of whether the SMS-coach was turned on or off (Figure MA1.2). Participants can view the messages (unique and all previous sent messages) by clicking on the messages under “text messages” in the personal home screen, see figure MA1.2

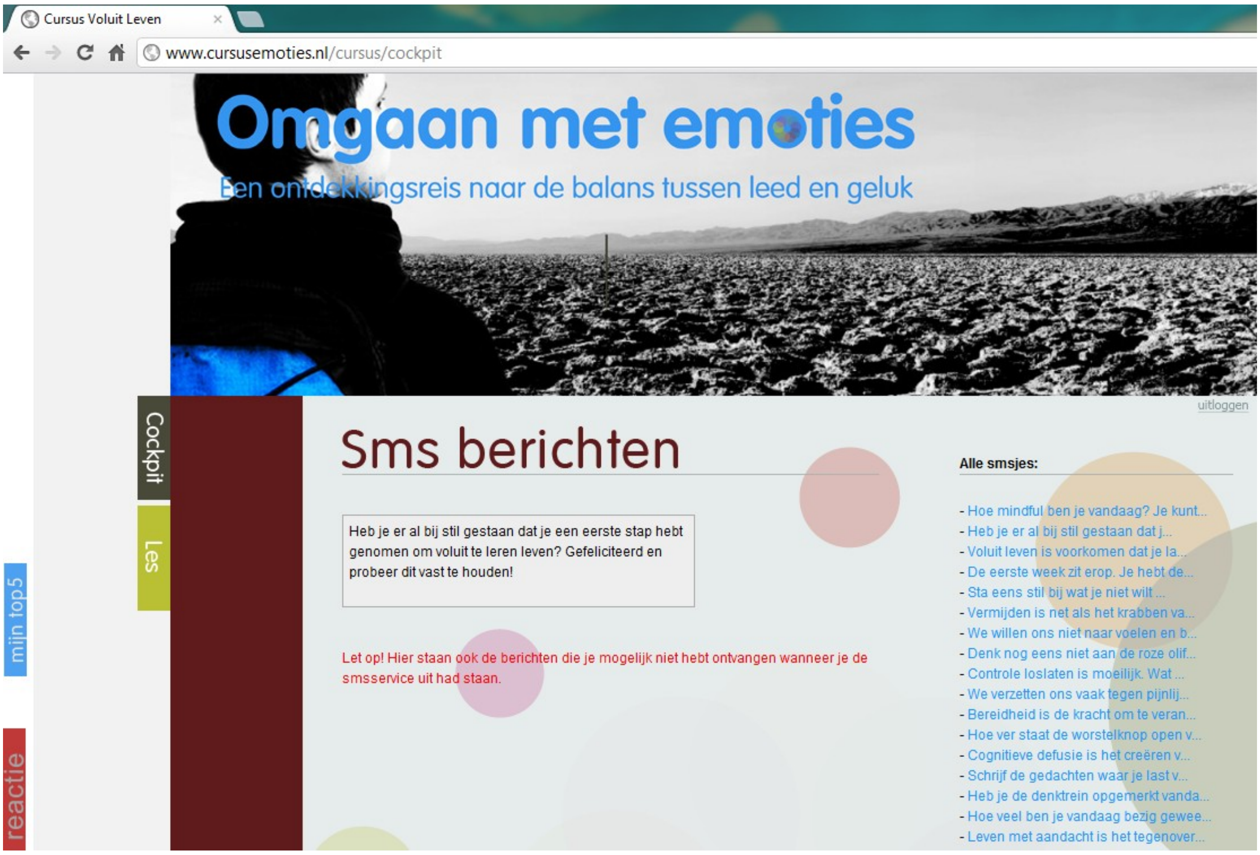

Figure MA1.2 – Text messages (and overview of all text message sent)

Movies

In 8 of the 9 lessons, a short movie (for example 3 minutes) was added in which the writer of the course or an experienced clinical psychologist explains the key points of the lesson. The movie does not contain other information than the text, but the information is presented in a different way (Figure MA1.3). Participants can click on the movies that are embedded in the lessons; they can view unique movies and previous movies.

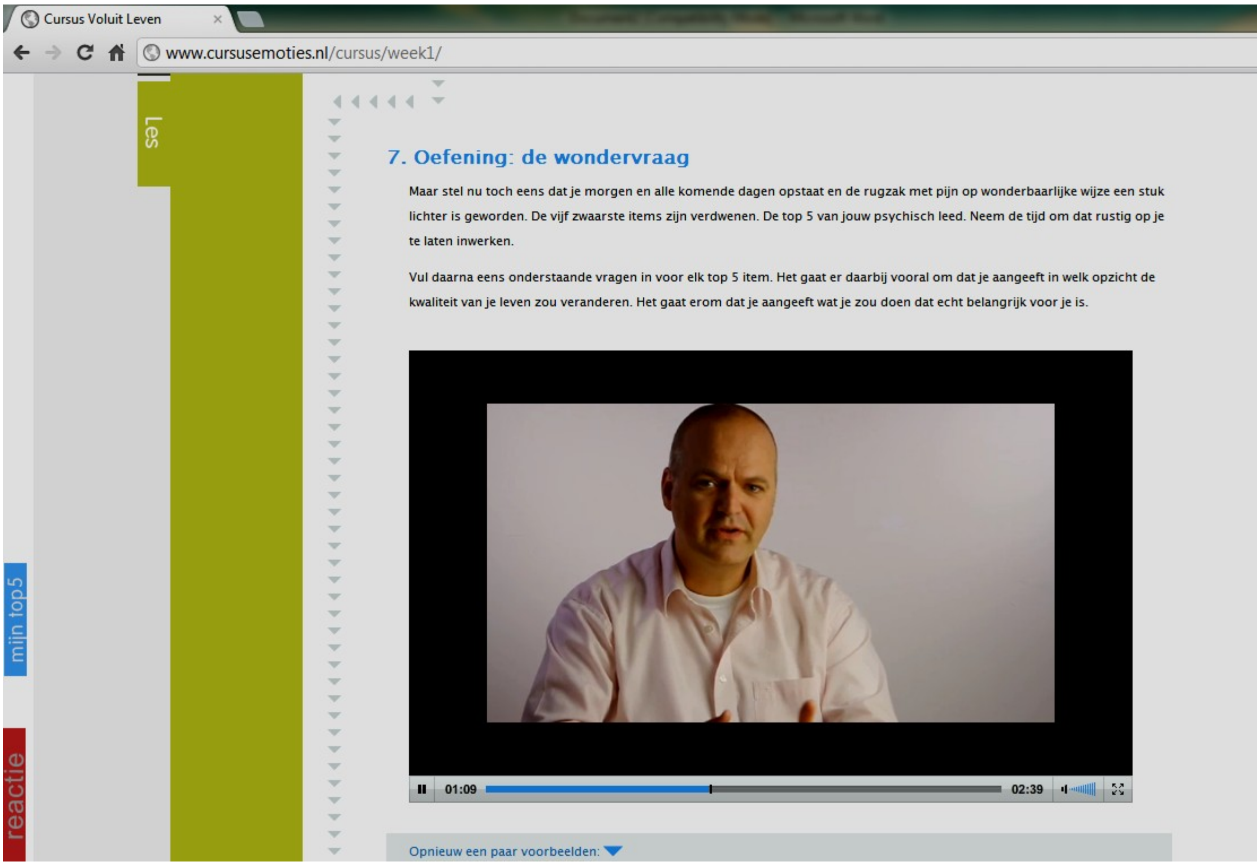

Figure MA1.3 – A movie (explanation of exercise)

Success stories

The intervention contained a success story for each of the lessons of the intervention that came available at the same time as the lessons. The participants accessed these stories from the cockpit, under 'experiences of others' and the stories were fictional, but based on the experiences of participants in an earlier study on the self-help book version of the intervention (Figure MA1.4). Participants can click on 'success stories' on the home screen to view unique stories and all previous sent stories during the course.

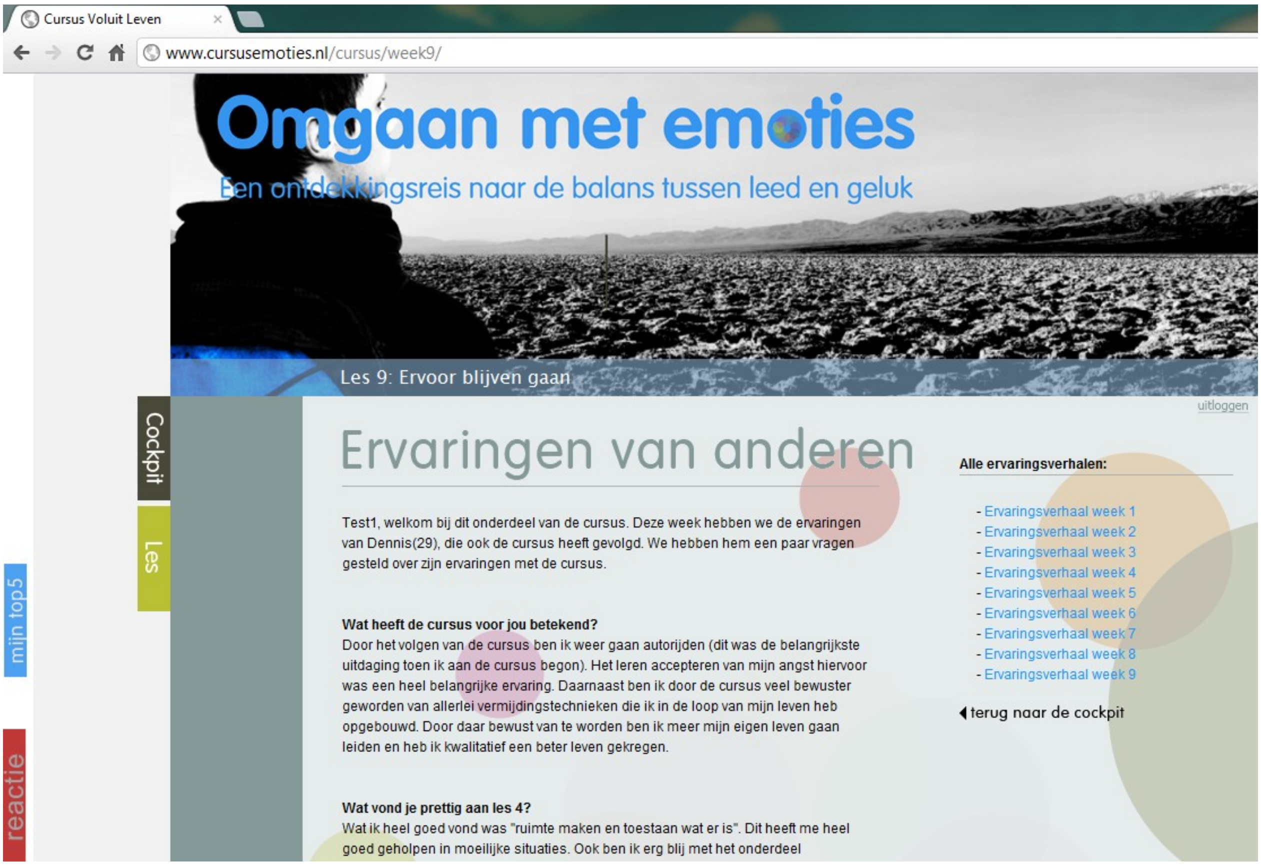

Figure MA1.4 – Example of a success story (lesson 9; text of story; possibility to view other stories (week 1-9))

Mindfulness

Participants can start a mindfulness exercise by clicking on the exercise, read or listen the information, and they can download the exercise for practicing, see figure MA1.5 . They can click on unique Mindfulness exercises as well as previous Mindfulness exercises.

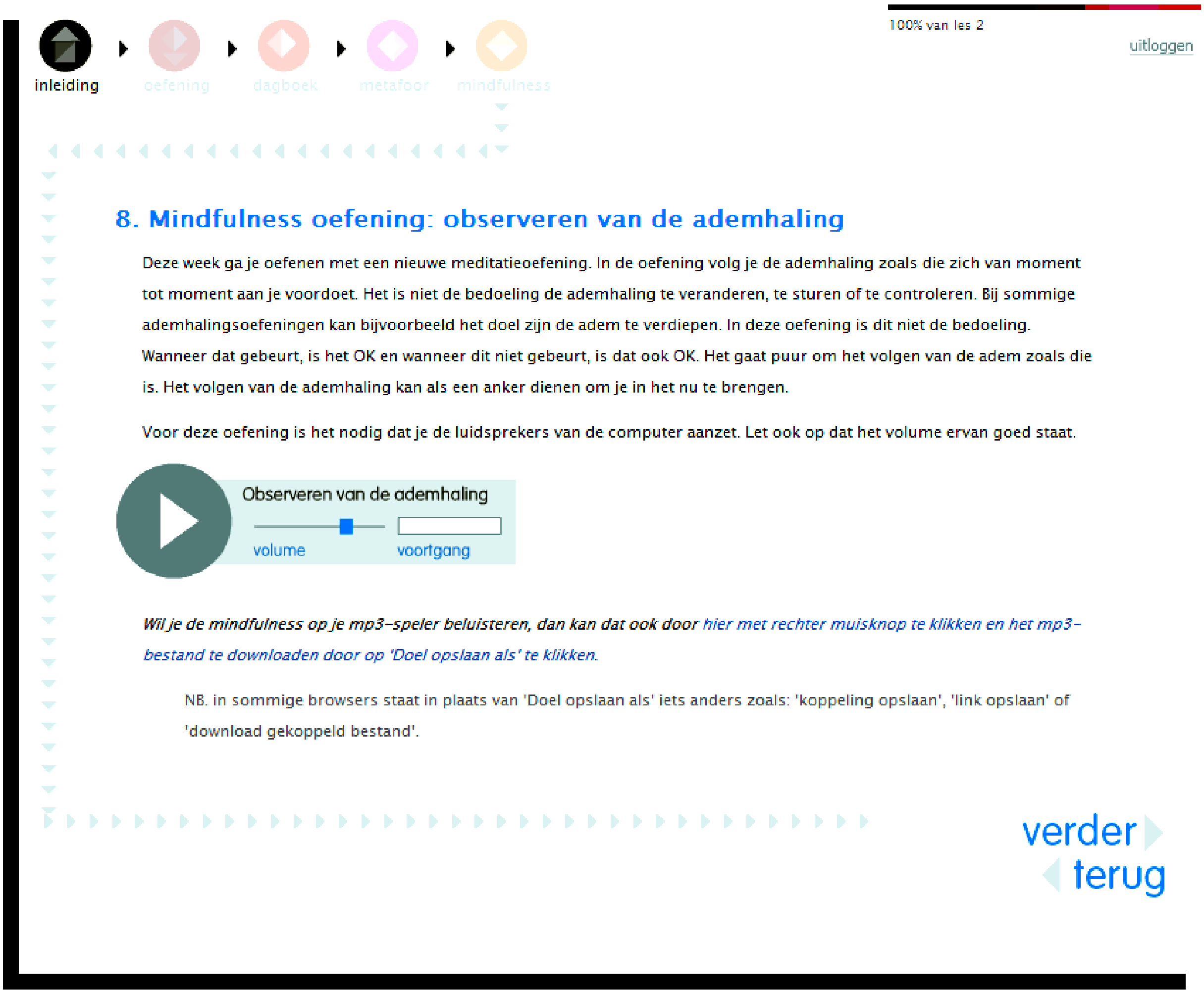

Figure MA1.5 – Example of a Mindfulness Exercise (read, listen to assignment, download exercise on mp3)
